# The cost of typhoid illness in low- and middle-income countries, a scoping review of the literature

**DOI:** 10.1371/journal.pone.0305692

**Published:** 2024-06-25

**Authors:** Frederic Debellut, Alena Friedrich, Ranju Baral, Clint Pecenka, Emmanuel Mugisha, Kathleen M. Neuzil

**Affiliations:** 1 Center for Vaccine Innovation and Access, PATH, Geneva, Switzerland; 2 University of Oregon, Eugene, OR, United States of America; 3 Center for Vaccine Innovation and Access, PATH, Seattle, WA, United States of America; 4 Center for Vaccine Innovation and Access, PATH, Kampala, Uganda; 5 Center for Vaccine Development and Global Health, University of Maryland School of Medicine, Baltimore, MD, United States of America; Virginia Commonwealth University, UNITED STATES

## Abstract

Typhoid fever is responsible for a substantial health burden in low- and middle-income countries (LMICs). New means of prevention became available with the prequalification of typhoid conjugate vaccines (TCV) by the World Health Organization (WHO) in 2018. Policymakers require evidence to inform decisions about TCV. The economic burden related to typhoid fever can be considerable, both for healthcare providers and households, and should be accounted for in the decision-making process. We aimed to understand the breadth of the evidence on the cost of typhoid fever by undertaking a scoping review of the published literature. We searched scientific databases with terms referring to typhoid fever cost of illness to identify published studies for the period January 1st 2000 to May 24^th^ 2024. We also conferred with stakeholders engaged in typhoid research to identify studies pending completion or publication. We identified 13 published studies reporting empirical data for 11 countries, most of them located in Asia. The total cost of a typhoid episode ranged from $23 in India to $884 in Indonesia (current 2022 United States Dollar [USD]). Household expenditures related to typhoid fever were characterized as catastrophic in 9 studies. We identified 5 studies pending completion or publication, which will provide evidence for 9 countries, most of them located in Africa. Alignment in study characteristics and methods would increase the usefulness of the evidence generated and facilitate cross-country and regional comparison. The gap in evidence across regions should be mitigated when studies undertaken in African countries are published. There remains a lack of evidence on the cost to treat typhoid in the context of increasing antimicrobial resistance. Decision-makers should consider the available evidence on the economic burden of typhoid, particularly as risk factors related to antimicrobial resistance and climate change increase typhoid risk. Additional studies should address typhoid illness costs, using standardized methods and accounting for the costs of antimicrobial resistance.

## Introduction

Typhoid fever is responsible for 110,000 annual deaths and 9 million annual cases globally and this burden disproportionately affects low- and middle-income countries in Asia and Africa [1]. In 2018, the World Health Organization (WHO) recommended that typhoid conjugate vaccines (TCV) be introduced in countries where typhoid is endemic and in countries with a high burden of drug resistant typhoid, to reduce typhoid burden [2]. At the end of 2023, five countries (Liberia, Malawi, Nepal, Pakistan, Zimbabwe) had introduced TCV into their routine immunization programs with support from Gavi, the Vaccine Alliance. Samoa also introduced TCV into its national vaccination program [3]. The vaccine was used in three additional countries to respond to outbreaks (Fiji, Pakistan, Zimbabwe).

The burden of typhoid is underestimated and often not well recognized as symptoms can be confused with other diseases such as malaria [4]. Diagnostic challenges are an additional barrier to properly account for burden [5]. Typhoid is often detected clinically and treated without proper testing as diagnostics are often not available and lack high sensitivity and specificity [6]. The associated economic burden is also not well documented [7]. The cost to treat patients presents a burden to the health system and to households. As drug resistant strains spread, typhoid is likely to become more costly [8].

Understanding the economic burden, or cost of illness, associated with typhoid fever is one of the critical elements to inform disease control policy. These data are also a key input to cost-effectiveness analyses, which likewise inform policy. With TCV now available to help alleviate typhoid burden, decision-makers require evidence on a number of factors, including the cost to treat typhoid cases, which provides insights into what savings TCV can yield. However, no review of the evidence on the cost of typhoid illness is currently available. To provide these important parameters for decision-making, and support global roll out of TCV, we performed a scoping review of the existing and forthcoming evidence on the cost of typhoid fever illness in all age groups.

## Materials and methods

### Search method

To identify existing evidence on cost of typhoid illness, we searched Pubmed and Web of Science/CABi Global Health/Proquest databases using search terms “typhoid fever,” “enteric fever,” “cost,” “cost of illness,” “economic burden,” “healthcare utilization,” “out of pocket costs,” “treatment costs,” and “household expenses,” for a period spanning January 1^st^ 2000 to May 24^th^ 2024, without applying any language restrictions. The main criteria for inclusion was that the study reported on empirical cost of typhoid illness. Studies were excluded if they did not report empirical cost of typhoid illness, if they reported data from high-income countries, if they focused on other topic areas such as vaccination, burden of disease, disease surveillance, epidemiology, diagnostics or animals, or if they were a duplicate found in one of the other databases. Screening of article titles and abstracts was performed by 2 researchers (FD and RB). When the title and abstract were not sufficient to assess inclusion or in cases of disagreement between the 2 researchers, the full article text was reviewed and a third researcher (CP) assisted with reaching final agreement.

To supplement the literature search, we contacted stakeholders involved in typhoid research work to identify cost of illness studies currently being implemented or completed but not yet published. Stakeholders included researchers involved in typhoid fever surveillance activities, or typhoid conjugate vaccines clinical trials or impact studies from a number of non-governmental and international organizations (International Vaccine Institute, Sabin Vaccine Institute, International Vaccine Access Center at Johns Hopkins Bloomberg School of Public Health), from the Academia (Center for Vaccine Development at the University of Maryland, Oxford Vaccine Group at University of Oxford, Malawi Liverpool Welcome Trust Research Program), and the donor community (Bill and Melinda Gates Foundation, Gavi the Vaccine Alliance).

### Data extraction and currency conversion

We did not perform data extraction for unpublished studies. For published studies, data extraction was done by a fourth researcher (AF) using EXCEL^®^. In addition to the main study characteristics, cost data related to typhoid fever were extracted in the category as reported in the original article, along with the perspectives used for reporting. When cost was reported for different age groups, we extracted and reported the different sets of cost data along with the population of interest and sample size. Cost data reported in the original study were adjusted and reported in 2022 USD units. To adjust the currency, cost estimates in the original article were first converted to the local currency unit (LCU) for the given year, then inflated to 2022 LCU using country specific annual inflation rates. The inflation adjusted LCU’s were then converted to 2022 USD. We used the official World Bank (WB) exchange rates to convert the LCU to USD, and the WB gross domestic product deflator values to adjust for inflation [9].

We report the cost outcomes according to standard cost of illness categories including direct medical costs (or cost directly related to the patient care e.g. cost for laboratory testing, drugs, hospital stay), direct non-medical costs (e.g. transportation, food), and indirect costs (or cost not directly related to patient care e.g. loss of productivity). When average direct medical costs were reported separately for different perspectives (i.e. provider and households) we summed the values to determine the total direct medical cost. When median direct medical costs were reported separately for different perspectives, we provide separate data points, highlighting the respective perspective. When no distinction was possible between cost categories, we reported values for a larger category (e.g. direct cost encompassing direct medical and direct non-medical cost).

We report findings descriptively, per country, per age group when available, and for 4 main categories: cost related to a typhoid case irrespective of the setting where care took place, cost related to a typhoid case treated in outpatient setting, cost related to a typhoid case treated in an inpatient setting, and finally, cost of typhoid intestinal perforations. We also searched all articles for cost of drug resistant typhoid and, when available, report on cost for each.

## Results

The search returned a total of 699 articles that were considered for inclusion in the review. Two additional published articles were included, identified through discussions with stakeholders involved in typhoid research work, because of their relevance despite not being initially identified by our search. Following the outreach and screening, a total of 13 articles were included in the review. A PRISMA diagram available in S1 Fig presents the total number of titles, abstracts and articles that matched the search criteria. The list of articles included in the review as well as their focal countries are available in S1 Table.

**Fig 1:**
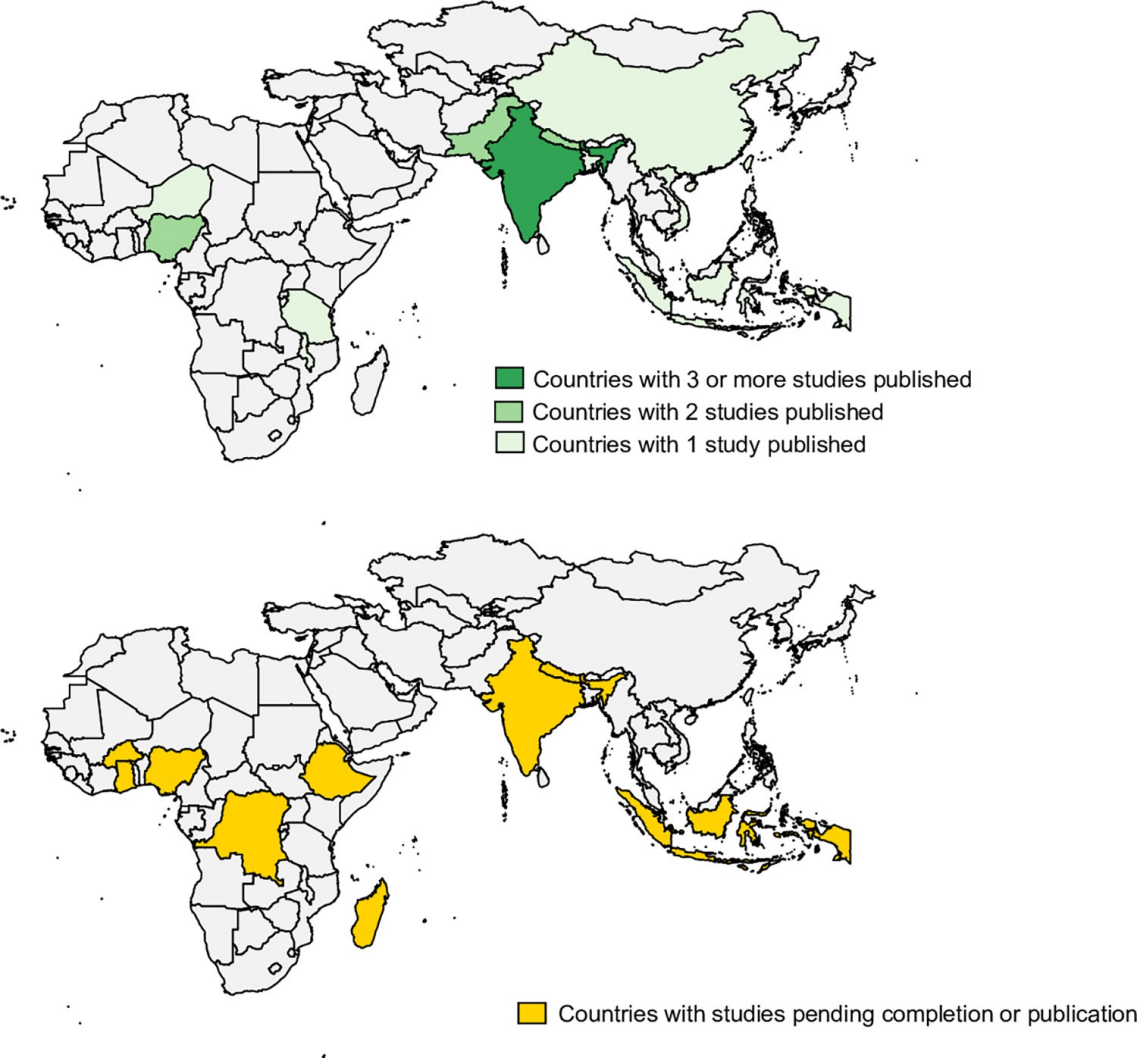
Existing evidence on cost of typhoid fever per country.

During the study period, a total of 13 studies have been published that report empirical data on the cost of typhoid fever in 11 countries. More than 60% of these studies are from the Asian continent whereas the remainder (40%, 4 studies) provide data from the African continent. The countries for which data are available, as well as the number of studies that have been published per country, are displayed in Fig 1.

### Studies pending completion or publication

We identified a total of five studies that included nine countries and either have ongoing implementation or were completed and results are pending publication. The majority of study countries are located on the African continent. Evidence is anticipated for Burkina Faso, Ghana, Ethiopia, and Madagascar from a multicenter study exploring the cost of illness and long-term socioeconomic impact of typhoid fever. This study was undertaken as part of the Severe Typhoid Fever in Africa Program implemented by the International Vaccine Institute (IVI) [10]. Another project led by IVI, the Typhoid Conjugate Vaccine Introduction in Africa program (THECA) aims to provide evidence for Nigeria and the Democratic Republic of Congo. Additional studies in Navi Mumbai, India by IVI and the US Centers for Disease Control and Prevention (CDC), and in the Kathmandu Valley in Nepal by Johns Hopkins University and Oxford University are completed and pending publication. Finally, a study from Universitas Gadjah Mada looked at direct and indirect costs of typhoid fever in five provinces of Indonesia. Countries for which data are anticipated are displayed in Fig 1.

### Settings and samples for published studies

The perspective and type of cost reported across published studies varied. Study characteristics are available in Table 1. The naming of study perspectives and cost categories were variable across studies. Only 1 study reported on a narrow healthcare provider perspective [11] while all others included a broader perspective covering costs borne by the healthcare provider and by patients and caregivers, or a societal perspective. The majority of studies report on direct medical costs, direct non-medical costs, and indirect costs. However, some studies do not separate direct medical and direct non-medical cost from the patient or household perspective. Some studies combine direct non-medical and indirect costs. It should also be noted that direct medical costs are supported by the provider in some studies, by households in others, and finally by both the provider and households in others, as represented in Table 1.

**Table 1:** Characteristics of published typhoid cost of illness studies.

Study #	Countries included	Setting	Study perspective	Total study sample	Sub-sample for which cost is reported	Direct medical costs	Direct non-medical costs	Indirect costs	Outcomes reported		
									Typhoid fever	Inpatient care	Outpatient care
Balh et al. (2004) [15]	India	Urban slum	Societal	223	0–2 y.o.	✓	✓	✓	✓	✓	✓
					2–5 y.o.						
					5–19 y.o.	(P)			BC+		
					Adults						
Sur et al. (2009) [11]	India	Hospitals in urban slum	Provider	83	Children	✓			✓	✓	✓
									BC+		
					Adults	(P)			Widal+		
Poulos et al. (2011) [12]	China, India, Indonesia, Pakistan, Vietnam	Urban, rural, slums, settlements	Public (paid by the government) and private (paid by households)	327	Children	✓	✓	✓	✓	✓	✓
					Adults	(B)			BC+	Not for all countries	Not for all countries
						(H) for China					
Riewpaiboon et al. (2014) [14]	Tanzania	Rural island	Societal	17	Children	✓		✓	✓		
					Adults	(H)			BC+		
Kaljee et al. (2017) [13]	Nepal	Urban	Societal	22	N/A	✓	✓	✓	✓	✓	✓
						(B)			BC+		
Mejia et al. (2020) [18]	Bangladesh	Urban	Healthcare provider, patients & caregivers	1,772	N/A	✓	✓	✓	✓	✓	✓
						(B)			BC+		
Mejia et al. (2020) [19]	Nepal	Urban & peri-urban	Healthcare provider, patients & caregivers	395	N/A	✓	✓	✓	✓	✓	✓
						(B)			BC+		
Mejia et al. (2020) [20]	Pakistan	Urban	Healthcare provider, patients & caregivers	1,029	N/A	✓	✓	✓	✓	✓	✓
						(B)			BC+		
Seyi-Olajide et al. (2020) [21]	Nigeria	Urban	Households	32	N/A	✓			✓	✓	
						(H)					
Wabada et al. (2020) [22]	Nigeria	Urban	Households	95	N/A	✓			✓	✓	
						(H)					
Adamou H. Et al. (2021) [23]	Niger	Rural and urban	Households	2931	N/A	✓			✓	✓	
						(H)					
Kumar et al. (2021) [17]	India	Rural and urban	Households	1,165	Patients from tertiary care hospital vs lower-level hospitals	✓	✓	✓	✓		✓
						(H)					
Limani et al. (2022) [16]	Malawi	Urban	Healthcare provider, households	109	Children	✓	✓	✓	✓	✓	✓
					Adults	(B)					

BC+: blood culture confirmed; y.o.: years old; N/A: not applicable; (P): provider perspective; (H): Households perspective; (B): Both provider and households’ perspectives

Indirect costs were valued using the human capital approach calculating the value of income loss due to typhoid illness or caring for someone sick with typhoid. Most studies valued the income loss by multiplying the number of days of work missed by an average or median daily salary. Average or median daily salaries used were often self-reported by patients and/or caregivers. Patients’ loss of income was only accounted for in adult patients. Only one study monetized children’s loss of school days [12]. One study did not provide sufficient details on how the calculation of indirect costs was determined [13].

A majority of studies reported on both outpatient and inpatient care in addition to providing an overall cost of treating a typhoid case, regardless of the setting. One study [14] bundled estimates together for both categories preventing report per setting of care. Studies looking specifically at typhoid related complications such as perforations reported only on inpatient care.

Five studies reported on costs for sub-samples, such as children, adults, or different age categories [11, 12, 14–16]. Only one study provided estimates for a sub-sample defined by the location of care (tertiary hospital vs lower level rural and urban hospitals) [17].

A majority of studies characterized typhoid related illness costs as catastrophic, using a variety of definitions. Four studies defined cost as catastrophic when the cost exceeded 10% or 15% of the annual household’s income [12, 18–20]. Three studies defined cost as catastrophic when the cost exceeded 40% of the monthly or annual nonfood household’s expenditure [16, 17, 21]. Two studies characterized cost as catastrophic without providing a definition [14, 23].

### Cost of typhoid illness

The reported cost estimates varied across countries, subpopulations, and care setting. The average total cost of a typhoid episode reported, irrespective of the setting or level of care where cases are treated, ranged from $23 in India to $270 in Indonesia. When looking at sub-populations for which data were reported, the range was even larger, from an average of $17 in children in India to an average of $456 in adults in Indonesia. The share of direct medical cost ranged from 14% of total cost in Tanzania to 88% in Bangladesh. When included, the share of indirect costs ranged from a low 10% of total cost in India to a high of 83% in Tanzania.

### Outpatient setting

For all age cases treated in outpatient care, the average total cost of typhoid illness ranged from $20 in India to $143 in China. For cases treated in outpatient settings, direct medical costs represented a large share of the total cost, from 65% in Malawi to 88% in Bangladesh. The share of indirect costs in the total cost ranged from 11% in Nepal to 34% in Malawi.

### Inpatient setting

For all age cases treated in an inpatient setting, the lowest ($201) and highest ($976) average total cost of typhoid illness were reported from 2 studies in India. When accounting for sub-populations for which cost data were reported, the highest total cost reported for inpatients increased to $1,095 for adults in Indonesia.

**Fig 2:**
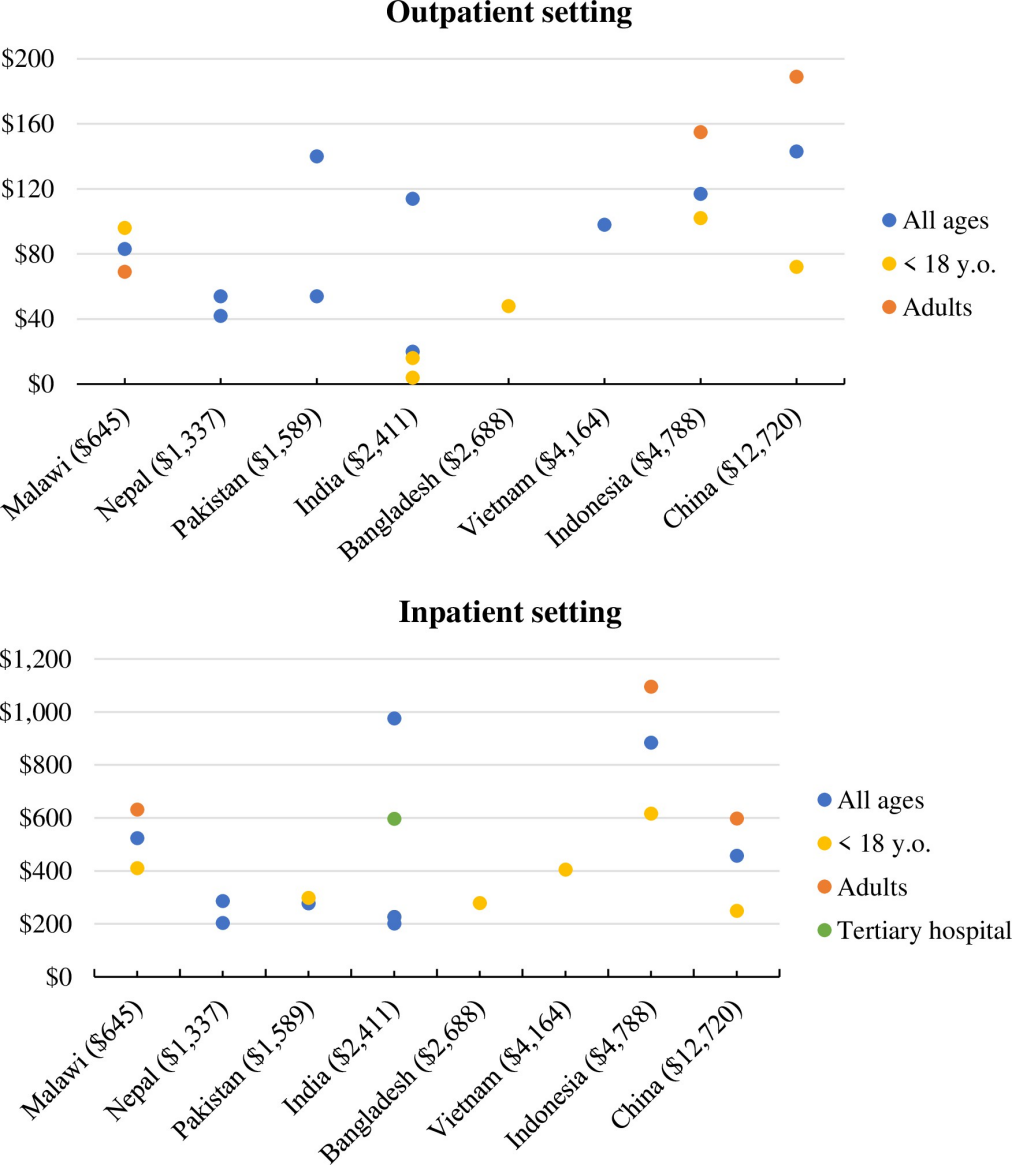
Average total cost of treating a typhoid case in inpatient and outpatient settings. Values in brackets show 2022 Growth Domestic Product per capita [24]; y.o.: years old.

Despite one study reporting a higher cost for children treated in outpatient settings, all other studies reporting estimates for children and adults showed a lower cost associated with children. Average total cost per setting is displayed in Fig 2.

For studies reporting both outpatient and inpatient costs of typhoid illness, the average cost to treat a case of typhoid was always higher in an inpatient setting. The outpatient cost represented, on average, 21% of the inpatient cost, ranging between 7% in India and 50% in Pakistan.

Only 4 studies reported specifically on the cost to treat ileal perforations. The total cost to treat an ileal perforation ranged from $551 in Niger to $1,735 in India. Direct medical costs for an ileal perforation ranged from $263 to $467 in Nigeria.

Only one study provided specific cost for antimicrobial resistant typhoid [20]. Drug resistant typhoid is commonly characterized as multi-drug resistant (MDR), defined as resistance to ampicillin, trimethoprim-sulfamethoxazole, and chloramphenicol, or extremely drug-resistant (XDR) defined as resistance to chloramphenicol, ampicillin, trimethoprim-sulfamethoxazole, fluoroquinolones as well as third-generation cephalosporins [25]. The study reports a median cost of illness for patients with XDR typhoid fever in Pakistan of US $223, characterizing it as higher than the median cost witnessed for all patients enrolled in the study but lower than all patients enrolled with nontraumatic ileal perforation. The same study provides an average cost for MDR enteric fever. No other study provides cost for MDR enteric fever or MDR typhoid fever. All cost data reported are available in Table 2.

**Table 2:** Reported cost of typhoid data per country and cost category.

Country and study reference	Population	Sample size for reported total cost estimate	Total cost	Direct medical	Direct non-medical	Indirect
**Cost of typhoid case treated in outpatient or inpatient setting**						
Bangladesh* [18]	<18 y.o.	1,500	$69	$61	$6	$24
*Patients & caregivers*						
*Healthcare provider*	N/A	N/A		$63		
China [12]		58	$268	$215		$55
	<18 y.o.	23	$143	$121		$21
	Adults	35	$353	$277		$77
Indonesia [12]		107	$270	$178		$92
	<18 y.o.	73	$153	$106		$47
	Adults	34	$456	$280		$176
India [15]		98	$194	$100	$35	$43
	<2 y.o.	3	$97	$36	$62	
	2–5 y.o.	33	$213	$149	$64	
	5–19 y.o.	51	$189	$87	$102	
	Adults	9	$179	$17	$161	
India [11]		83		$38		N/A
	<18 y.o.	77		$31		N/A
India [12]		79	$23	$16		$8
	<18 y.o.	54	$17	$12		$5
Nepal [12]		22	$152	$113		$39
Nepal* [19]		332	$64	$40	$5	Patient $51
*Patients & caregivers*						Care giver $20
*Healthcare provider*	N/A	N/A		$86		
Pakistan [12]	<18 y.o.	66	$78	$67		$13
Pakistan* [20]		796	$147	$122	$4	Patient $90
*Patients & caregivers*						Care giver $24
*Healthcare provider*	N/A	N/A		$10 - $46		
Tanzania [14]		17	$190	$27	$6	$157
	<15 y.o.	n.a.	$226			
	Adults	n.a.	$165			
Vietnam [12]	<18 y.o.	17	$183	$170		$13
**Cost of typhoid case treated in outpatient setting**						
Bangladesh* [18]	<18 y.o.	1,084	$48	$42	$3	$21
*Patients & caregivers*						
China [12]		58	$143			
	<18 y.o.	23	$72			
	Adults	35	$189			
Indonesia [12]		107	$117			
	<18 y.o.	73	$102			
	Adults	34	$155			
India [15]		98	$114			
India [11]	<18 y.o.	67		$4		
India [12]		79	$20			
	<18 y.o.	54	$16			
Malawi [16]		65	$83	$54	$1	$28
	<18 y.o.	42	$96	$56	$2	$38
	Adults	23	$69	$52	$1	$16
Nepal [12]		n.a.	$54	$48		$6
Nepal* [19]		242	$42	$29	$4	Patient $45
*Patients & caregivers*						Care giver $8
Pakistan [12]	<18 y.o.	66	$54			
Pakistan* [20]		773	$140	$117	$4	Patient $90
*Patients & caregivers*						Care giver $24
Vietnam [12]	<18 y.o.	17	$98			
**Cost of typhoid case treated in inpatient setting**						
Bangladesh* [18]	<18 y.o.	416	$279	$209	$58	$27
*Patients & caregivers*						
China [12]		58	$457			
	<18 y.o.	23	$249			
	Adults	35	$598			
Indonesia [12]		107	$884			
	<18 y.o.	73	$616			
	Adults	34	$1,095			
India [15]		11	$976			
India [11]		16		$182		
	<18 y.o.	10		$212		
	Adults	6		$133		
India [12]		79	$201			
	<18 y.o.	54	$226			
India [17]	Patients from tertiary care hospital	221	$597	$368	$53	Patient $54
						Care giver $122
	Patients from lower level hospitals	782	$204	$110	$20	Patient $26
						Care giver $48
Malawi [16]		44	$524	$398	$49	$77
	<18 y.o.	21	$410	$310	$37	$63
	Adults	23	$632	$483	$60	$89
Nepal [12]		n.a.	$286	$204		$82
Nepal* [19]		90	$204	$150	$18	Patient $81
						Care giver $36
*Patients & caregivers*						
Pakistan [12]	<18 y.o.	66	$298			
Pakistan* [20]		23	$278	$266	$4	Patient $0
*Patients & caregivers*						Care giver $30
Vietnam [12]	<18 y.o.	17	$405			
**Cost of treating typhoid related nontraumatic ileal perforations**						
India [17] **†**		110	$1,735	$1,320		$415
Niger [23]		2,931	$551			
Nigeria [21]	< 15 y.o.	32		$467		
Nigeria [22]	< 15 y.o.	95		$263		
Pakistan* [20] **†**		123	$451	$202	$49	Patient $294
*Patients & caregivers*						Care giver $92

* Indicates values reported as median; y.o.: year olds; n.a.: not available

** indicates we report the cost for severe enteric fever rather than cost for salmonella typhi because of the additional details provided for severe enteric fever (only direct medical cost is provided for salmonella typhi)

**†**indicates studies reporting on cost of nontraumatic ileal perforation due to enteric fever rather than typhoid.

## Discussion

This scoping review presents evidence from existing cost of typhoid illness studies for a total of 11 countries, mostly from Asia while upcoming evidence should provide data for additional countries, a majority of those located in Africa. This body of evidence highlights the significant economic burden typhoid fever represents.

The published studies used a variety of methods and data are not always reported systematically. Alignment in the study perspective and naming and defining of cost categories would greatly increase the usefulness of the evidence generated and facilitate cross-country and regional comparison [26, 27]. While these methodological differences between studies largely prevent direct comparison, they illustrate the cost to treat typhoid for specific countries and offer trends for Asia and Africa so that decision-makers understand the potential magnitude of the cost of typhoid fever and implications for prevention and control.

When considering direct medical costs, there is often less detail on household expenditures. In contrast, direct medical costs from the provider perspective are typically better delineated with details about costs of laboratory testing and diagnostics, costs for drugs/pharmacy, and other costs related to facilities or inpatient stay or staff costs. It should be noted, however, that household expenditures related to typhoid fever were characterized as catastrophic in 9 out of the 13 studies, highlighting the substantial economic burden a typhoid episode represents for populations living in LMICs.

The sample size for most studies was rather small, which limits the use and interpretation of the estimates from such studies. The challenge is more poignant when looking at sub-samples of the population.

The underrepresentation of countries from the African region in the published studies to date highlights a gap that should be filled in the coming years with the publication of studies covering 6 countries in the region. It’s likely that more studies for this region will be beneficial as typhoid burden in this setting has historically been underestimated and under studied.

The evidence provided by the identified studies represents quite a range of cost estimates, at times with large ranges in the same country. This is likely caused by a number of factors such as severity of cases treated, geographic area, level of hospital where cases are treated, difficulties with diagnostics, misdiagnosis, delayed treatment seeking, etc. The study in India by Kumar et al. (2021) reporting costs per level of hospital is a good example of how the cost can increase in higher, tertiary level hospitals compared to lower level hospitals [17].

This scoping review includes some limitations. While we have not included any language restrictions in our database search, we are aware that journals publishing in other languages than English may not be indexed in the databases used. As an example, we identified an article in French through discussions with partners. We only report on published articles and did not search the grey literature, however, additional evidence that has not been published may be available from this source. Finally, we elected to discuss with partners involved in typhoid research to identify ongoing studies or studies completed and pending publications. While we have tried for this process to be as comprehensive as possible, we may have overlooked studies implemented by partners with whom we were unable to discuss.

This scoping review provided an opportunity to identify current gaps in the scientific evidence. In addition to variable reporting practices among individual papers, there is a lack of data on cost by severity of disease or level of treatment. As importantly, despite for one datapoint in Pakistan, there is a lack of data on the cost of drug resistant typhoid. Repeating studies in some of the older study settings and/or with enough detail to identify first and subsequent lines of drugs to treat typhoid could be a way to explore the potential increasing cost of treating typhoid in a context of increasing drug resistance. Further, it is important to differentiate costs in children and adults. As Gavi support for typhoid conjugate vaccines ends at 15 years of age, the cost of implementing pediatric and adult vaccine programs will vary considerably in Gavi-eligible countries. Evidence on the economic burden related to children is likely of higher interest to decision-makers in these countries. Implementing additional cost of illness studies, coupled with surveillance to detect different resistant typhoid strains could also inform our understanding of the potentially increasing economic burden of typhoid fever.

Decision-makers should account for the available evidence on the economic burden of typhoid when making decisions on the utilization of typhoid disease prevention and control interventions, including typhoid conjugate vaccines and improvement to water and sanitation infrastructure. The studies described in this paper illustrate the costs of typhoid illness, and more studies may further illuminate the burden of typhoid in additional countries and account for drug resistance trends. The economic burden related to typhoid fever is described as catastrophic for households in a large proportion of countries where data are available Decision makers should, thus, consider investments in typhoid prevention and control.

## Supporting information

S1 Checklist Preferred Reporting Items for Systematic reviews and Meta-Analyses extension for Scoping Reviews (PRISMA-ScR) checklist. (DOCX)

S1 File Search terms. (PDF)

S1 Fig PRISMA diagram. (TIF)

S1 Table Articles included in the review. (PDF)
