# Supplementary material for: The cost of typhoid illness in low- and middle-income countries, a scoping review of the literature
Source: PLoS One. 2024 Jun 25;19(6):e0305692. doi: 10.1371/journal.pone.0305692 (PMC11198801; doi:10.1371/journal.pone.0305692)
Supplement: S1 File — (PDF) [file pone.0305692.s002.pdf]

Search terms

typhoid fever: "typhoid fever"[MeSH Terms] OR ("typhoid"[All Fields] AND "fever"[All Fields]) OR "typhoid fever"[All Fields]

OR

enteric fever: "typhoid fever"[MeSH Terms] OR ("typhoid"[All Fields] AND "fever"[All Fields]) OR "typhoid fever"[All Fields] OR ("enteric"[All Fields] AND "fever"[All Fields]) OR "enteric fever"[All Fields]

AND

cost: "economics"[Subheading] OR "economics"[All Fields] OR "cost"[All Fields] OR "costs and cost analysis"[MeSH Terms] OR ("costs"[All Fields] AND "cost"[All Fields] AND "analysis"[All Fields]) OR "costs and cost analysis"[All Fields]

typhoid fever: "typhoid fever"[MeSH Terms] OR ("typhoid"[All Fields] AND "fever"[All Fields]) OR "typhoid fever"[All Fields]

OR

enteric fever: "typhoid fever"[MeSH Terms] OR ("typhoid"[All Fields] AND "fever"[All Fields]) OR "typhoid fever"[All Fields] OR ("enteric"[All Fields] AND "fever"[All Fields]) OR "enteric fever"[All Fields]

AND

cost of illness: "cost of illness"[MeSH Terms] OR ("cost"[All Fields] AND "illness"[All Fields]) OR "cost of illness"[All Fields]

typhoid fever: "typhoid fever"[MeSH Terms] OR ("typhoid"[All Fields] AND "fever"[All Fields]) OR "typhoid fever"[All Fields]

OR

enteric fever: "typhoid fever"[MeSH Terms] OR ("typhoid"[All Fields] AND "fever"[All Fields]) OR "typhoid fever"[All Fields] OR ("enteric"[All Fields] AND "fever"[All Fields]) OR "enteric fever"[All Fields]

AND

economic burden: "financial stress"[MeSH Terms] OR ("financial"[All Fields] AND "stress"[All Fields]) OR "financial stress"[All Fields] OR ("economic"[All Fields] AND "burden"[All Fields]) OR "economic burden"[All Fields]

typhoid fever: "typhoid fever"[MeSH Terms] OR ("typhoid"[All Fields] AND "fever"[All Fields]) OR "typhoid fever"[All Fields]

OR

enteric fever: "typhoid fever"[MeSH Terms] OR ("typhoid"[All Fields] AND "fever"[All Fields]) OR "typhoid fever"[All Fields] OR ("enteric"[All Fields] AND "fever"[All Fields]) OR "enteric fever"[All Fields]

AND

healthcare: "delivery of health care"[MeSH Terms] OR ("delivery"[All Fields] AND "health"[All Fields] AND "care"[All Fields]) OR "delivery of health care"[All Fields] OR "healthcare"[All Fields] OR "healthcare's"[All Fields] OR "healthcares"[All Fields]

utilization: "statistics and numerical data"[Subheading] OR ("statistics"[All Fields] AND "numerical"[All Fields] AND "data"[All Fields]) OR "statistics and numerical data"[All Fields] OR "utilization"[All Fields] OR "utilisation"[All Fields] OR "utilisations"[All Fields] OR "utilise"[All Fields] OR "utilised"[All Fields] OR "utilises"[All Fields] OR "utilising"[All Fields] OR "utilities"[All Fields] OR "utility"[All Fields] OR "utilizations"[All Fields] OR "utilize"[All Fields] OR "utilized"[All Fields] OR "utilizer"[All Fields] OR "utilizers"[All Fields] OR "utilizes"[All Fields] OR "utilizing"[All Fields]

typhoid fever: "typhoid fever"[MeSH Terms] OR ("typhoid"[All Fields] AND "fever"[All Fields]) OR "typhoid fever"[All Fields]

OR

enteric fever: "typhoid fever"[MeSH Terms] OR ("typhoid"[All Fields] AND "fever"[All Fields]) OR "typhoid fever"[All Fields] OR ("enteric"[All Fields] AND "fever"[All Fields]) OR "enteric fever"[All Fields]

AND

out of pocket costs: "health expenditures"[MeSH Terms] OR ("health"[All Fields] AND "expenditures"[All Fields]) OR "health expenditures"[All Fields] OR ("out"[All Fields] AND "pocket"[All Fields] AND "costs"[All Fields]) OR "out of pocket costs"[All Fields]

typhoid fever: "typhoid fever"[MeSH Terms] OR ("typhoid"[All Fields] AND "fever"[All Fields]) OR "typhoid fever"[All Fields]

OR

enteric fever: "typhoid fever"[MeSH Terms] OR ("typhoid"[All Fields] AND "fever"[All Fields]) OR "typhoid fever"[All Fields] OR ("enteric"[All Fields] AND "fever"[All Fields]) OR "enteric fever"[All Fields]

AND

treatment costs: "health care costs"[MeSH Terms] OR ("health"[All Fields] AND "care"[All Fields] AND "costs"[All Fields]) OR "health care costs"[All Fields] OR ("treatment"[All Fields] AND "costs"[All Fields]) OR "treatment costs"[All Fields]

typhoid fever: "typhoid fever"[MeSH Terms] OR ("typhoid"[All Fields] AND "fever"[All Fields]) OR "typhoid fever"[All Fields]

OR

enteric fever: "typhoid fever"[MeSH Terms] OR ("typhoid"[All Fields] AND "fever"[All Fields]) OR "typhoid fever"[All Fields] OR ("enteric"[All Fields] AND "fever"[All Fields]) OR "enteric fever"[All Fields]

AND

household: "family characteristics"[MeSH Terms] OR ("family"[All Fields] AND "characteristics"[All Fields]) OR "family characteristics"[All Fields] OR "household"[All Fields] OR "households"[All Fields] OR "household's"[All Fields] OR "householder"[All Fields] OR "householder's"[All Fields] OR "householders"[All Fields]

expenses: "expense"[All Fields] OR "expenses"[All Fields] OR "expensive"[All Fields] OR "expensively"[All Fields]
