# Supplementary figures and images for: The cost of typhoid illness in low- and middle-income countries, a scoping review of the literature
Source: PLoS One. 2024 Jun 25;19(6):e0305692. doi: 10.1371/journal.pone.0305692 (PMC11198801; doi:10.1371/journal.pone.0305692)

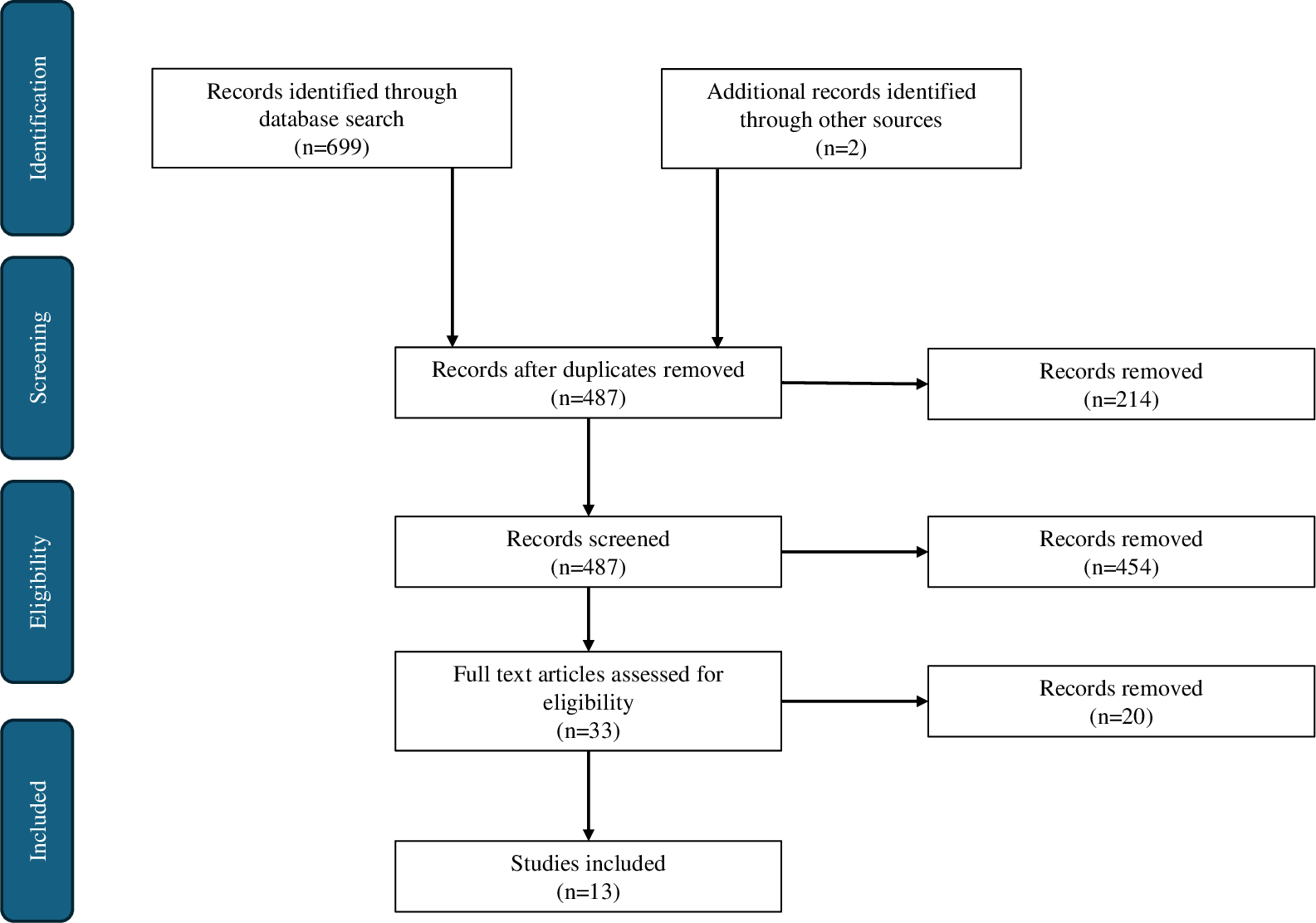

Supplement: S1 Fig — (TIF) [file pone.0305692.s003.tif]
