# Supplementary material for: The cost of typhoid illness in low- and middle-income countries, a scoping review of the literature
Source: PLoS One. 2024 Jun 25;19(6):e0305692. doi: 10.1371/journal.pone.0305692 (PMC11198801; doi:10.1371/journal.pone.0305692)
Supplement: S1 Table — (PDF) [file pone.0305692.s004.pdf]

Supplementary table 1: Articles included in the review

Year of publication	Author	Title	Country	Reference
2004	Balh et al.	Costs of Illness Due to Typhoid Fever in an Indian Urban Slum Community: Implications for Vaccination Policy	India	[1]
2009	Sur et al.	Treatment Cost for Typhoid Fever at Two Hospitals in Kolkata, India	India	[2]
2011	Poulos et al.	Cost of illness due to typhoid fever in five Asian countries	China, India, Indonesia, Pakistan, Vietnam	[3]
2014	Riewpaiboon et al.	Cost of Illness Due to Typhoid Fever in Pemba, Zanzibar, East Africa	Tanzania	[4]
2017	Kaljee et al.	Social and Economic Burden Associated With Typhoid Fever in Kathmandu and Surrounding Areas: A Qualitative Study	Nepal	[5]
2020	Mejia et al.	Typhoid and Paratyphoid Cost of Illness in Bangladesh: Patient and Health Facility Costs From the Surveillance for Enteric Fever in Asia Project II	Bangladesh	[6]
2020	Mejia et al.	Typhoid and Paratyphoid Cost of Illness in Nepal: Patient and Health Facility Costs From the Surveillance for Enteric Fever in Asia Project II	Nepal	[7]
2020	Mejia et al.	Typhoid and Paratyphoid Cost of Illness in Pakistan: Patient and Health Facility Costs From the Surveillance for Enteric Fever in Asia Project II	Pakistan	[8]
2020	Seyi-Olajide et al.	Catastrophic Healthcare Expenditure from Typhoid Perforation in Children in Nigeria	Nigeria	[9]
2020	Wabada et al.	The Socio-Economic Impact of Typhoid Intestinal Perforation in Children in a Developing Country	Nigeria	[10]
2021	Adamou H. Et al.	The burden of typhoid perforation of the small intestine in Niger.	Niger	[11]
2021	Kumar et al.	Cost of Illness Due to Severe Enteric Fever in India.	India	[12]
2022	Limani et al.	Estimating the economic burden of typhoid in children and adults in Blantyre, Malawi: a costing cohort study	Malawi	[13]

[1] Rajiv Bahl, Anju Sinha, Christine Poulos, et al. Costs of Illness Due to Typhoid Fever in an Indian Urban Slum Community: Implications for Vaccination Policy. J Health Popul Nutr. 2004 Sep;22(3):304-310. https://pubmed.ncbi.nlm.nih.gov/15609783/

[2] Dipika Sur, Susmita Chatterjee, Arthorn Riewpaiboon, et al. Treatment Cost for Typhoid Fever at Two Hospitals in Kolkata, India. J Health Popul Nutr. 2009 Dec;27(6):725-732. https://doi.org/10.3329%2Fjhpn.v27i6.4323

[3] Christine Poulos, Arthorn Riewpaiboon, John F. Stewart, et al. Cost of illness due to typhoid fever in five Asian countries. Trop Med Int Health. 2011 Mar;16(3):314-23. https://doi.org/10.1111/j.1365-3156.2010.02711.x

[4] Arthorn Riewpaiboon, Moritz Piatti, Benedikt Ley, et al. Cost of Illness Due to Typhoid Fever in Pemba, Zanzibar, East Africa. J Health Popul Nutr. 2014 Sep;32(3):377-385. http://www.ncbi.nlm.nih.gov/pmc/articles/pmc4221443/

[5] Linda M. Kaljee, Alfred Pach, Denise Garrett, et al. Social and Economic Burden Associated With Typhoid Fever in Kathmandu and Surrounding Areas: A Qualitative Study. J Infect Dis. 2018 Nov 10;218(suppl_4):S243-S249. https://doi.org/10.1093/infdis/jix122

[6] Nelly Mejia, Sarah W. Pallas, Samir Saha, et al. Typhoid and Paratyphoid Cost of Illness in Bangladesh: Patient and Health Facility Costs From the Surveillance for Enteric Fever in Asia Project II. Clin Infect Dis. 2020 Dec 1;71(Suppl 3):S293-S305. https://doi.org/10.1093/cid/ciaa1334

[7] Nelly Mejia, Taiwo Abimbola, Jason R. Andrews, et al. Typhoid and Paratyphoid Cost of Illness in Nepal: Patient and Health Facility Costs From the Surveillance for Enteric Fever in Asia Project II. Clin Infect Dis. 2020 Dec 1;71(Suppl 3):S306-S318. https://doi.org/10.1093/cid/ciaa1335

[8] Nelly Mejia, Farah Qamar, Mohammad T. Yousafzai, et al. Typhoid and Paratyphoid Cost of Illness in Pakistan: Patient and Health Facility Costs From the Surveillance for Enteric Fever in Asia Project II. Clin Infect Dis. 2020 Dec 1;71(Suppl 3):S319-S335. https://doi.org/10.1093/cid/ciaa1336

[9] Justina O. Seyi-Olajide, Jamie Anderson, Augustine O. Enivwaene, et al. Catastrophic Healthcare Expenditure from Typhoid Perforation in Children in Nigeria. Surg Infect (Larchmt). 2020 Sep;21(7):586-591. https://doi.org/10.1089/sur.2020.134

[10] Samuel Wabada, Auwal Mohammed Abubakar. The Socio-Economic Impact of Typhoid Intestinal Perforation in Children in a Developing Country. Arch Pediatr 5: 174. DOI: 10.29011/2575-825X.100174.

[11] Adamou H, Amadou Magagi I, Adakal O, et al. The burden of typhoid perforation of the small intestine in Niger. Journal Sahélien des Sciences de la Santé – Vol. 001, N°2.

[12] Dilesh Kumar, Atul Sharma, Saroj Kumar Rana, et al. Cost of Illness Due to Severe Enteric Fever in India. J Infect Dis. 2021 Nov 15; 224(Suppl 5): S540–S547. https://doi.org/10.1093%2Finfdis%2Fjiab282

[13] Fumbani Limani, Christopher Smith, Richard Wachepa, et al. Estimating the economic burden of typhoid in children and adults in Blantyre, Malawi: A costing cohort study. PLoS One. 2022 Nov 23;17(11):e0277419. https://doi.org/10.1371/journal.pone.0277419
